# Supplementary material for: VprBP/DCAF1 Triggers Melanomagenic Gene Silencing through Histone H2A Phosphorylation
Source: Biomedicines. 2023 Sep 17;11(9):2552. doi: 10.3390/biomedicines11092552 (PMC10526264; doi:10.3390/biomedicines11092552)
Supplement: Supplementary file 1 [file biomedicines-11-02552-s001.zip › Combined supplementary file-Shin et al.pdf]

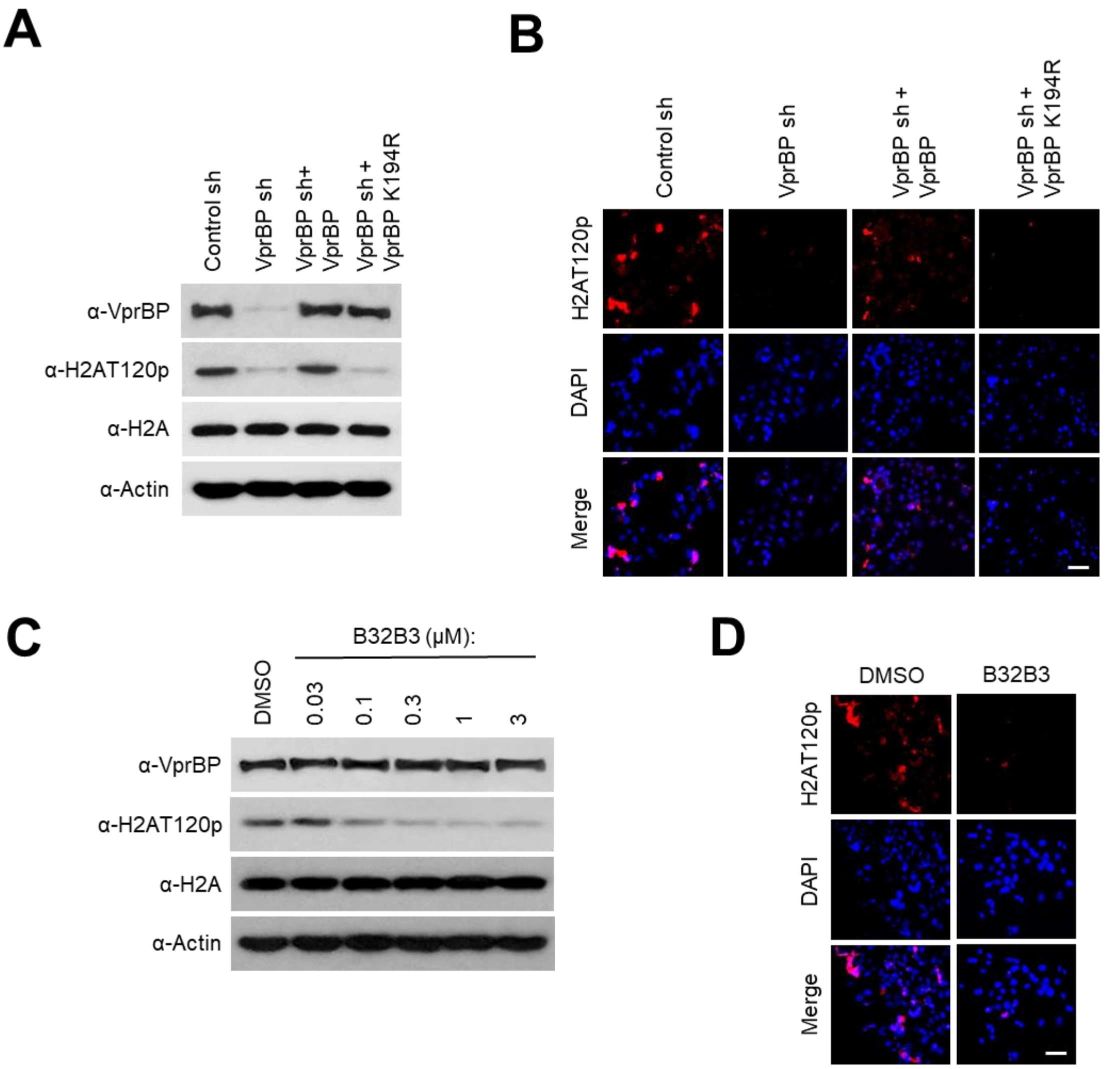

Figure S1. H2AT120p by VprBP in melanoma cells.

Western blotting (A and C) and immunostaining (B and D) analyses were performed as described in Fig. 1 but using MeWo cells depleted/rescued of VprBP (A and B) or treated with B32B3 (C and D). Bar, 10 μm.

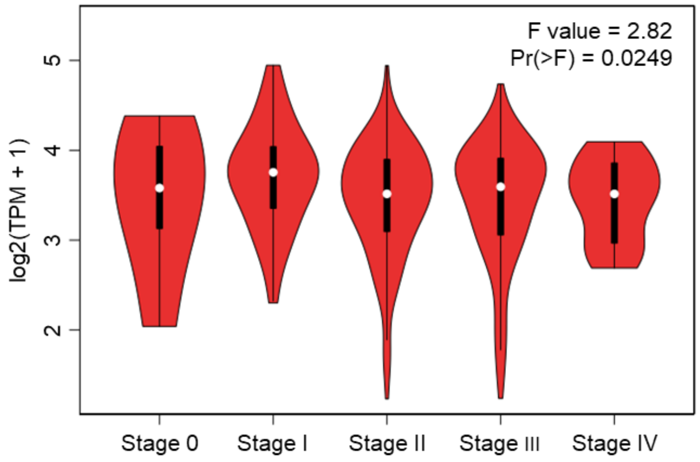

Figure S2. Violin plots of VprBP expression in different clinical stages of skin cutaneous melanoma (SKCM).

The expression levels of VprBP in patient samples were analyzed using the Gene Expression Profiling Interactive Analysis (GEPIA) online tool, which is available at http://gepia.cancer-pku.cn/.

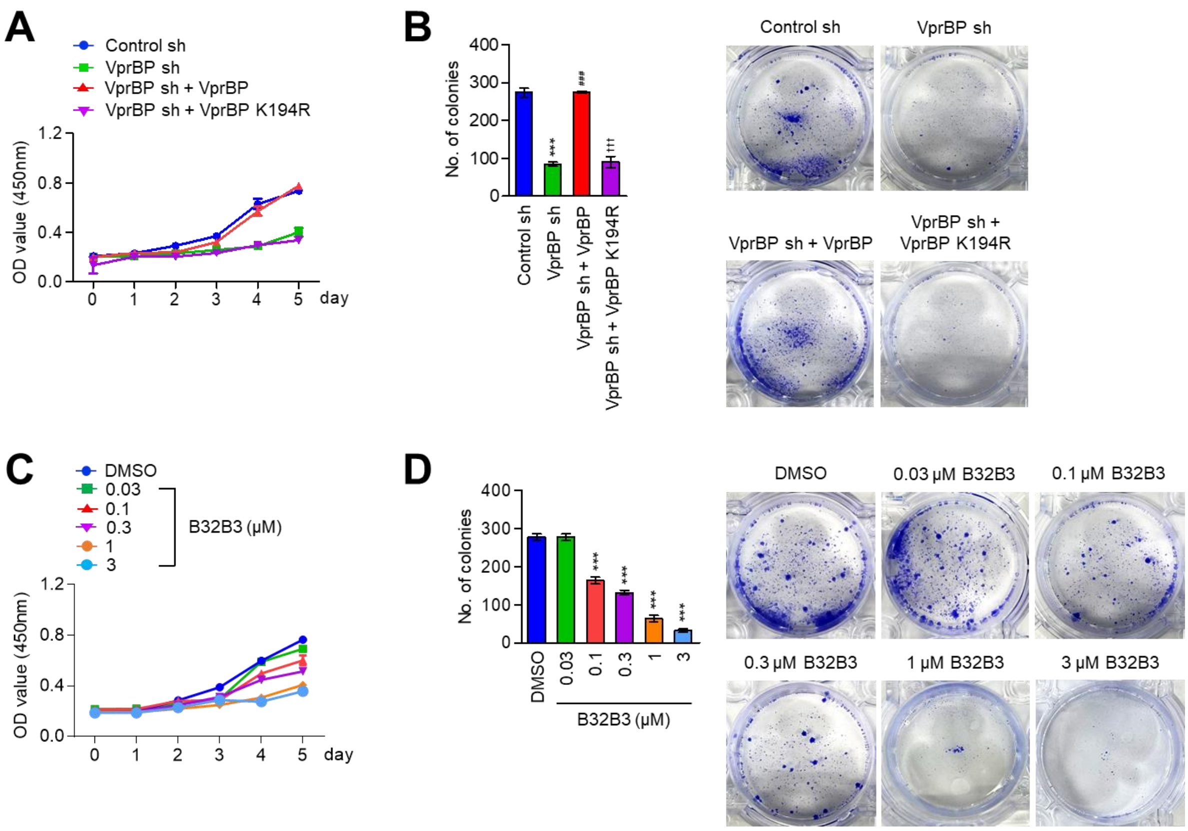

Figure S3. Preventive effects of VprBP downregulation on melanomagenesis.

Cell proliferation and colony formation assays were performed using VprBP-depleted/rescued (A and B) or VprBP inhibitor-treated (C and D) MeWo cells. (N=3). ***p < 0.001 versus control sh; ###P < 0.001 versus VprBP sh; †††P < 0.001 versus control sh; For (D): ***P<0.001 versus DMSO.

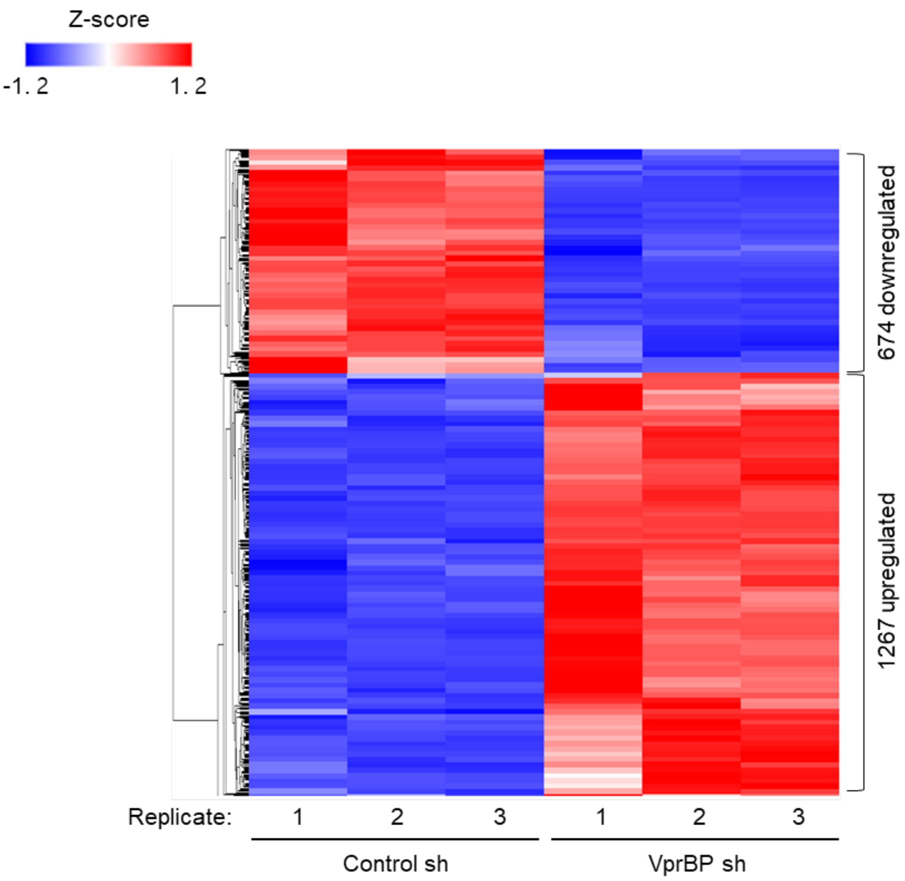

Figure S4. Heatmap representation of VprBP-responsive genes.

A heatmap shows 1267 upregulated genes and 674 downregulated genes in response to VprBP knockdown in G361 cells.

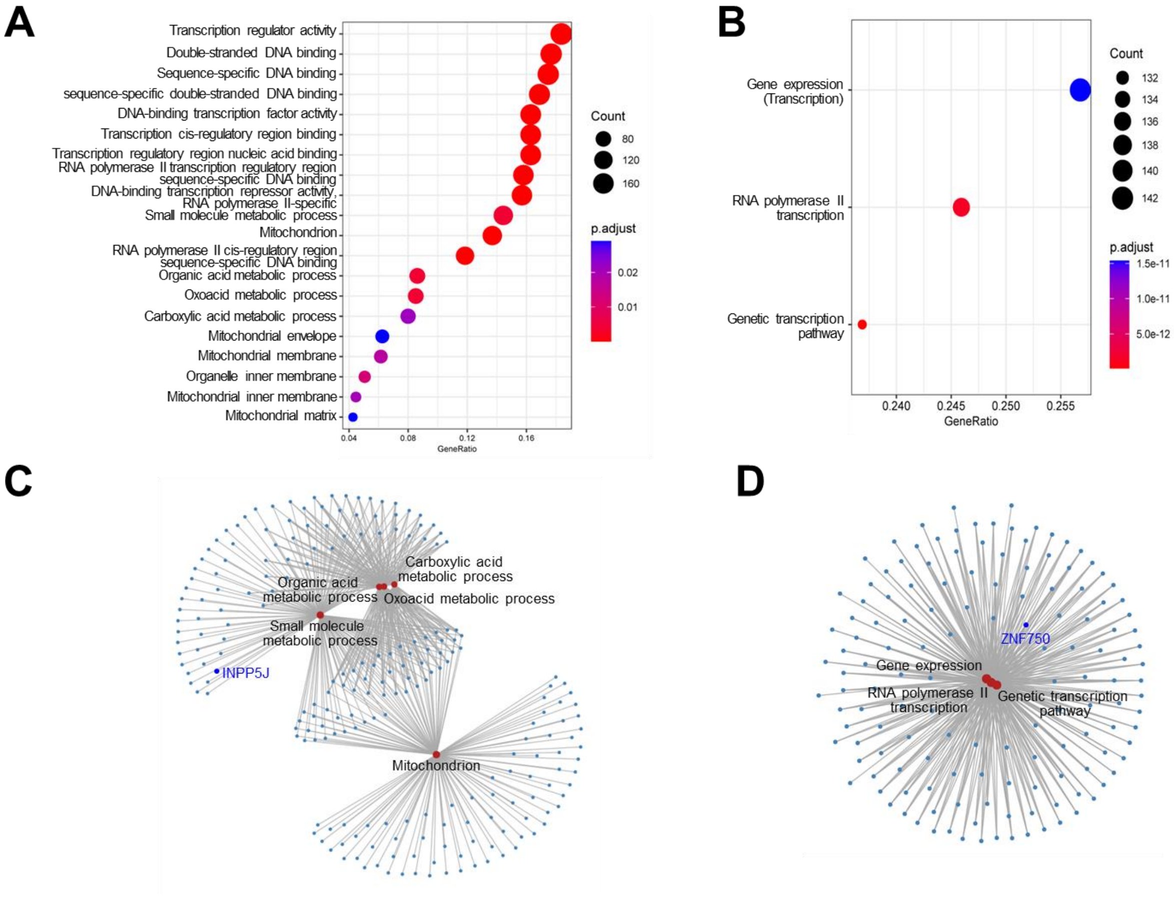

Figure S5. Gene ontology and Reactome pathway analysis of VprBP-responsive genes.

A, B Dot plot represents significantly enriched Gene Ontology (GO) terms **A** and Reactome pathways **B** for the 1267 up-regulated genes with a significance level of p-value < 0.05 and q-value <0.05. The size of dots on the plot indicates the number of enriched genes, while the color represents the enrichment significance in the pathway. The data were filtered with a Benjamini-Hochberg method with a significance threshold of both p-value and q-value <0.05 using a function implemented in the R package clusterProfiler 4.7.0. (See also Supplementary information 2)

C, D The gene-concept network indicates the connection between a gene and GO term (C) or Reactome pathway (D). Red dots in C and D indicate top-ranked enrichment terms shown in A and B.

A B

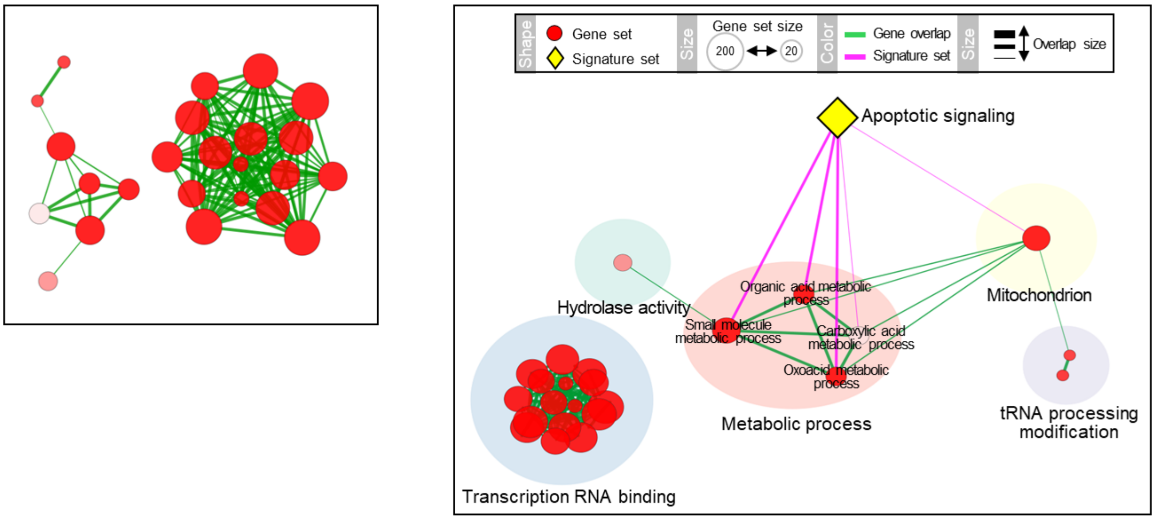

Figure S6. Network-based functional enrichment analysis of VprBP-responsive genes.

A The graphic represents the network-based enrichment map. To create a network enrichment map, we first determined the functional pathways using g:Profiler (https://biit.cs.ut.ee/gprofiler/gost). The g:Profiler results were used to generate the enrichment map with EnrichmentMap application (version 3.3.5) in Cytoscape software (version 3.9.1).

B The network clusters of gene sets (red dots) were annotated by AutoAnnotate software (ver. 1.4.0), with the overlap between each gene set indicated with green solid lines. To identify an association of gene sets to apoptotic signaling, the signature analysis was further performed using the post-analysis feature of Cytoscape. The yellow diamond represents the gene set of apoptotic signaling, and pink solid lines indicate overlap between the apoptotic signature genes and enriched gene sets. The data were scored using one-sided Fisher's Exact Test and overlaps passing the significance thresholds (nominal $p < 0.05$ and FDR < 0.05) were selected. (See also Supplementary information 2).

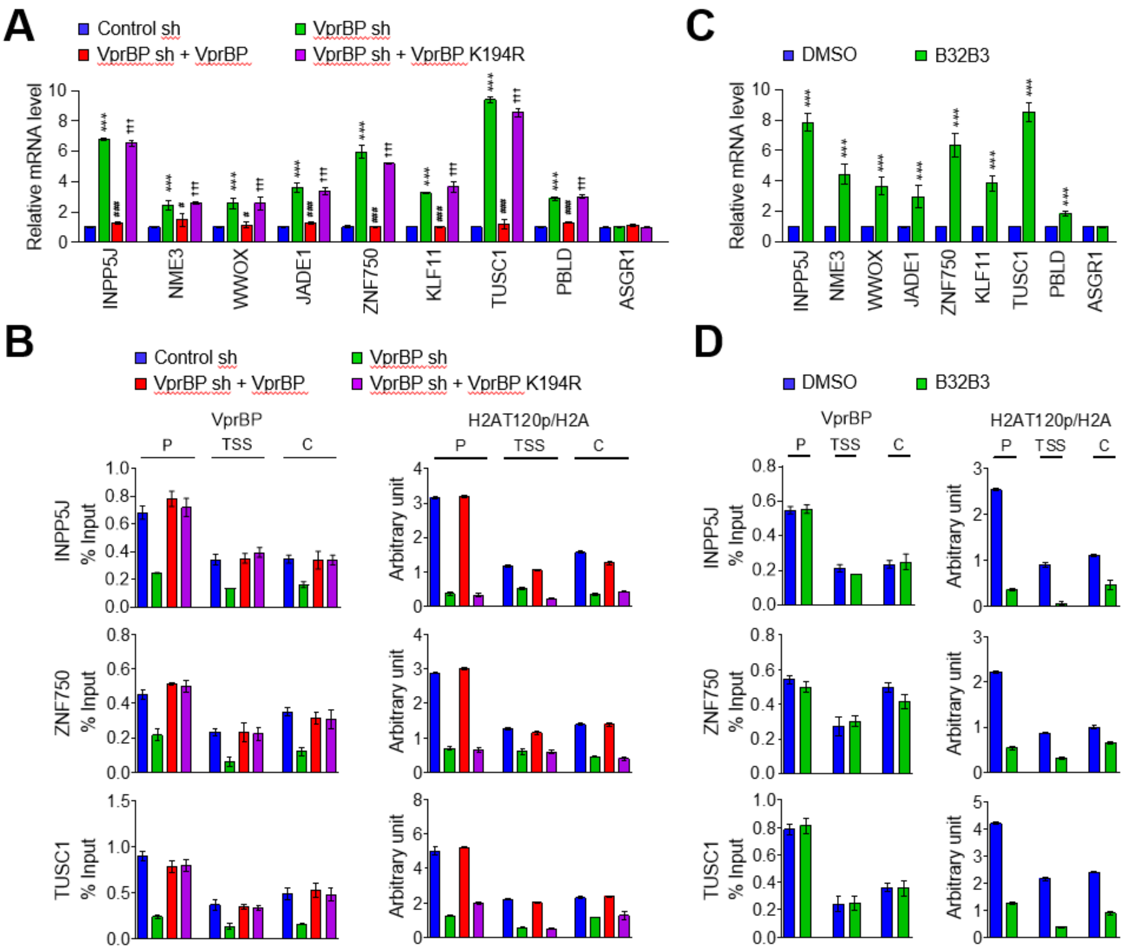

Figure S7. RT-qPCR and ChIP-qPCR analyses of VprBP target genes.

RT-qPCR (A and C) and ChIP-qPCR (B and D) analyses were conducted on representative target genes using VprBP-depleted/rescued (A and B) or B32B3-treated (C and D) MeWo cells. RT-qPCR data were expressed as mean ± S.D. (N=3). *P* values were calculated using paired *t*-test. ***p < 0.001 versus control sh; $^{\#}$P < 0.05, $^{\#\#\#}$P < 0.001 versus VprBP sh; †††P < 0.001 versus control sh; and ***P<0.001 versus DMSO.

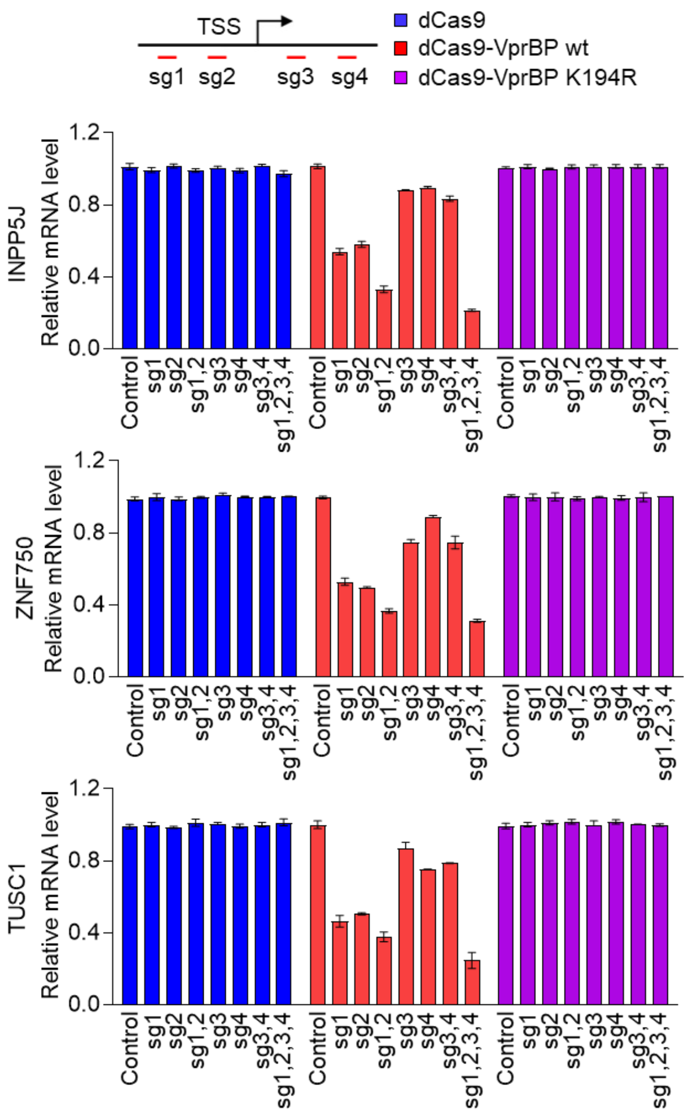

Figure S8. dCas9-VprBP-driven inactivation of target genes.

sgRNA and dCAS9 expression constructs (dCAS9, dCas9-VprBP wt or dCas9-VprBP K194R) were transiently transfected into G361 cells and qRT-PCR experiments were performed using sgRNAs specific for INPP5J, ZNF750, and TUSC1 genes as in Fig. 4.

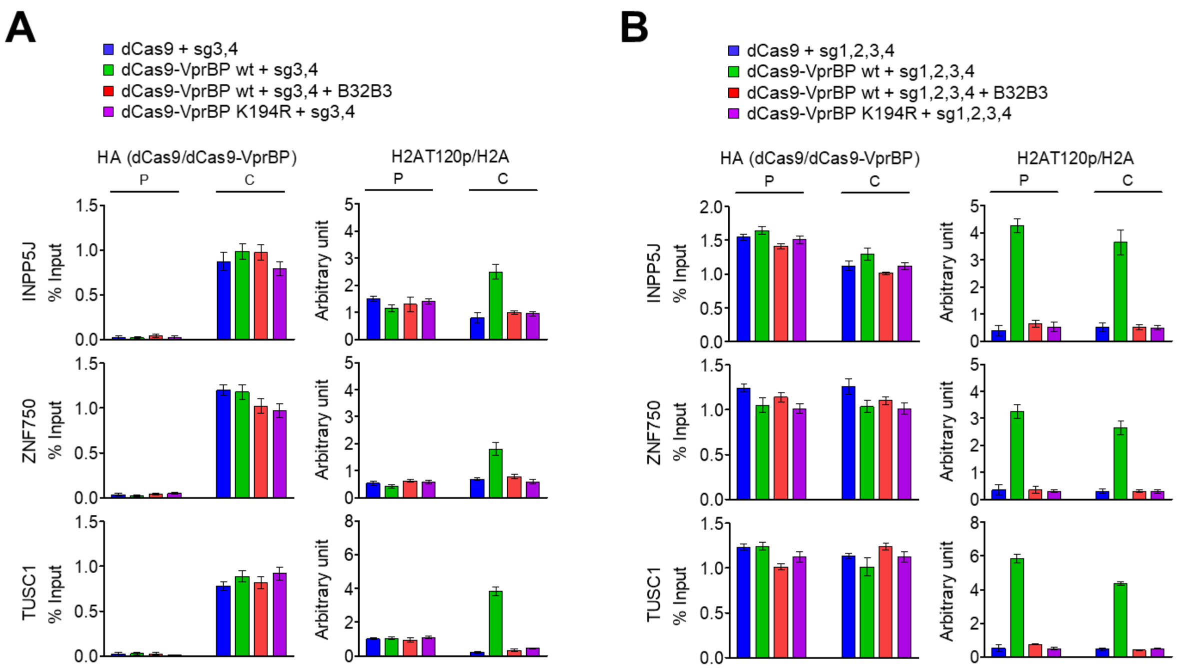

Figure S9. dCas9-VprBP-driven specific inhibition of the target gene.

A VprBP-depleted G361 cells were transfected with dCas9 and two sgRNA expression constructs as in Fig. 5C but using sgRNA 3 and 4 pair targeting coding regions. The levels of H2AT120p at the promoter and coding regions of the genes were assessed by ChIP-qPCR.

B ChIP assays were performed as in A but using sgRNA 1, 2, 3 and 4 together.

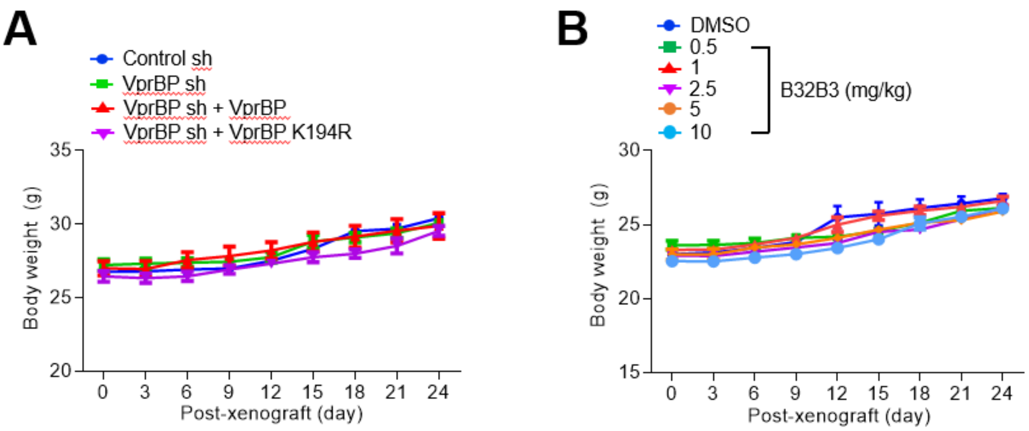

Figure S10. Body weight measurements of mice bearing VprBP-depleted or B32B3-treated melanoma xenografts.

A Melanoma xenograft body weights were measured every 3 days after injection of mock-depleted control, VprBP-depleted or wild-type/mutant VprBP-transfected VprBP-depleted MeWo cells.

B Melanoma xenograft body weights were measured every 3 days over a 24-day B32B3 treatment. Mean body weights (g) ± S.D are shown.

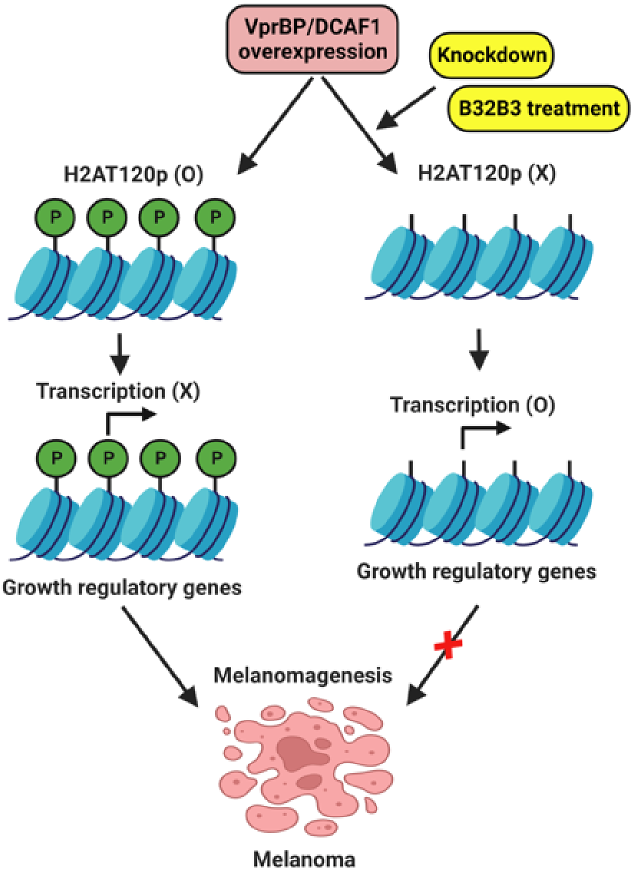

Figure S11. Summary of VprBP-induced melanomagenic gene silencing.
VprBP is recruited to growth regulatory genes for localized H2AT120p, and this phosphorylation mark establishes inactive chromatin environment to block transcriptional initiation. VprBP knockdown- or VprBP inhibitor-induced lockage of H2AT120p reactivates VprBP target genes and suppresses melanoma tumor growth. Diagram was created with BioRender.com.

Table S1. List of the primers used in RT-qPCR.

Primers	Forward (5'-3')	Reverse (5'-3')
INPP5J	CTGGGACTGGATCGGCTTAT	CGATGAGGATGCTGTGGTTG
NME3	GCTGACCATCTTCGCTAACC	ACCAACTTGAAGCCCTTCCT
WWOX	TCTGGGGCACTTCTACCTTG	GCATCGCCCAATAGTCGTTT
JADE1	GCAGCGATGCTACGACAATA	CATCAGGAGACTGGCAGACA
ZNF750	AAGACCCGAGACACTTCCTG	GAATCGGCAGGTTAGAGGGA
KLF11	GTATGAGCTCCTGGGGTCAA	ATGCACAGTGGTGGTGACAT
TUSC1	CCCGACTTGAGAAGCTGGAA	GGAGTCGGGTTCCTGTAGAG
PBLD	ACGGAGAATCTGCTGCAAGT	AACCCACGGTGCAAAATATC

Table S2. List of the primers used in ChIP-qPCR.

Primers	Forward (5'-3')	Reverse (5'-3')
INPP5J (P)	GACTGTCACCTCCAACTCCA	CAGTGGTTGTGGTGAAGGTG
INPP5J (TSS)	CTCAGCAGCCAGTTTGAAGG	GCCATTTCAACCTGCTGAGA
INPP5J (C)	GCTCAACATGGCCAAGAACA	AAGCTGGCTTCCGTTTCTTG
ZNF750 (P)	CGCCTTGCTCTCTTGAAGTC	ACAGCTTTACTCACCTGCCT
ZNF750 (TSS)	GGGAGTCGGCGAGATAACTT	ACCTGATATTCCTCCTGCCC
ZNF750 (C)	CCCGCCTCACTTTTACACAG	AGGAAGTGTCTCGGGTCTTG
TUSC1 (P)	CCTTTTGTCGCCCAGGATG	CCTTCCGGGTTCAAGAGACT
TUSC1 (TSS)	CTGAACAGCAAAGCACTCCC	CAAAGGAGGCCGGGAATTTG
TUSC1 (C)	CGCAGCCTCTTCCGTCAG	CTTCTCAAGTCGGGCCCTC

Table S3. Sequences of sgRNAs.

Genes	Sequences (5'- 3')
INPP5J (sg1)	CAAGCCCTTCGAGCTATCTG
INPP5J (sg2)	AAACACTCCCGTAGGCCTGT
INPP5J (sg3)	GCGCAGACATGATCGCCATA
INPP5J (sg4)	CCCTCATCCCGTGAACGCCG
ZNF750 (sg1)	ACCGCAAGAGCTGCGGAGCA
ZNF750 (sg2)	TCAAGCGCGCATACCCTGGG
ZNF750 (sg3)	TCGGGCGCCTCGGCGTTGTC
ZNF750 (sg4)	AGGCAGCCTCGACGGTGACG
TUSC1 (sg1)	TCGGTTCTGAGACAAAGCTA
TUSC1 (sg2)	CTCCGGAGTGCAACCACCAG
TUSC1 (sg3)	GCAGCAACTCCCGCGCCTAG
TUSC1 (sg4)	CATCCGTGCGCGGGACGAGT
