# Supplementary material for: VprBP/DCAF1 Triggers Melanomagenic Gene Silencing through Histone H2A Phosphorylation
Source: Biomedicines. 2023 Sep 17;11(9):2552. doi: 10.3390/biomedicines11092552 (PMC10526264; doi:10.3390/biomedicines11092552)
Supplement: Supplementary file 1 [file biomedicines-11-02552-s001.zip › Information S1-Shin et al.pdf]

Gene expression profiling

Gene name	FDR step up (KD vs Cont)	Fold change (KD vs Cont)
AKAP6	1.73E-08	9.37E+00
NKD1	4.00E-08	2.09E+01
SLC6A14	4.08E-08	1.81E+01
TEX9	5.15E-08	1.54E+01
MAFB	6.37E-08	1.82E+01
SULT1A1	6.95E-08	1.76E+01
ATP6V1B1	9.30E-08	1.70E+01
FZD4	9.69E-08	5.26E+00
PLEKHG4B	1.08E-07	8.06E+00
PLAA	1.09E-07	8.83E+00
PAIP2B	1.09E-07	5.26E+00
STK36	1.15E-07	6.86E+00
MINDY1	1.26E-07	6.55E+00
TBC1D30	1.35E-07	1.08E+01
HYLS1	1.44E-07	6.56E+00
FAAH	1.51E-07	4.87E+00
IL20RA	1.52E-07	7.01E+00
H2BC12	1.53E-07	7.00E+00
SLC25A45	1.71E-07	1.05E+01
DGCR6	1.71E-07	4.60E+00
ZNF32	1.73E-07	1.09E+01
SMPDL3B	1.74E-07	6.00E+00
EFNA5	1.78E-07	3.77E+00
NREP	1.87E-07	7.20E+00
PPFIBP2	1.87E-07	1.08E+01
TMEM156	1.91E-07	7.54E+00
CYP20A1	2.02E-07	1.23E+01
LYPD6B	2.02E-07	3.87E+00
HES6	2.11E-07	1.19E+01
SYNJ1	2.15E-07	7.00E+00
TESMIN	2.38E-07	1.06E+01
MAP2	2.48E-07	5.63E+00
MXD3	2.51E-07	5.05E+00
NQO2	2.53E-07	4.54E+00
TMEM139	2.58E-07	6.58E+00
ZNF778	2.64E-07	6.69E+00
CYP2J2	2.79E-07	5.27E+00
MPPE1	2.81E-07	3.38E+00

SCAMP5	2.90E-07	7.08E+00
RALGPS1	2.92E-07	4.87E+00
ENSG00000248538	2.94E-07	9.55E+00
HDHD3	3.22E-07	3.80E+00
INPP5J	3.28E-07	5.61E+00
MPZL3	3.31E-07	4.27E+00
GCDH	3.31E-07	3.68E+00
LRRC37A4P	3.38E-07	6.47E+00
SETD6	3.49E-07	2.39E+01
ALPK3	3.49E-07	7.35E+00
TMEM170B	3.57E-07	1.01E+01
ODC1	3.59E-07	5.56E+00
ZNF253	3.59E-07	7.36E+00
PKD1L2	3.59E-07	8.27E+00
ACCS	3.60E-07	4.80E+00
ERMAP	3.64E-07	4.38E+00
THYN1	3.67E-07	3.62E+00
DTWD2	3.70E-07	7.97E+00
C11orf54	3.73E-07	4.51E+00
L3MBTL2	3.73E-07	3.41E+00
SRR	3.80E-07	8.05E+00
FGFR4	3.86E-07	1.17E+01
VWA2	3.89E-07	6.87E+00
CENPV	3.90E-07	7.25E+00
TTC12	3.93E-07	8.88E+00
RAB24	3.95E-07	4.74E+00
EPB41L4A-AS1	3.98E-07	4.27E+00
CFAP410	4.00E-07	6.65E+00
BCKDHB	4.04E-07	3.63E+00
HDHD2	4.06E-07	6.28E+00
NEBL	4.09E-07	3.53E+00
RASSF1	4.16E-07	3.71E+00
KLF8	4.18E-07	3.13E+00
TMEM135	4.19E-07	3.63E+00
KRCC1	4.25E-07	4.34E+00
FAM117B	4.26E-07	4.00E+00
ENDOV	4.38E-07	4.30E+00
FAM171B	4.41E-07	3.45E+00
RWDD2B	4.45E-07	3.59E+00
PCDHA11	4.55E-07	8.47E+00

PER3	4.59E-07	5.09E+00
RPUSD4	4.60E-07	3.27E+00
RTKN2	4.88E-07	6.17E+00
SLC2A11	4.93E-07	5.31E+00
ABCG1	5.03E-07	6.99E+00
DENND1B	5.24E-07	3.21E+00
ZFP62	5.29E-07	3.92E+00
ZADH2	5.37E-07	4.08E+00
SMAD6	5.38E-07	5.22E+00
HCG11	5.53E-07	8.82E+00
TMEM144	5.64E-07	8.06E+00
C19orf54	5.75E-07	3.30E+00
PNPLA8	5.85E-07	3.79E+00
SLC25A14	5.85E-07	4.97E+00
S1PR2	5.96E-07	4.66E+00
EFS	6.00E-07	5.10E+00
BICDL2	6.23E-07	1.71E+01
KAZALD1	6.43E-07	2.43E+01
MTG1	6.45E-07	3.24E+00
ALDH6A1	6.53E-07	3.41E+00
FAM118B	6.53E-07	4.36E+00
DOCK11	6.68E-07	8.29E+00
ARHGAP33	6.93E-07	4.47E+00
NXPH4	7.04E-07	6.61E+00
ICA1	7.05E-07	1.95E+01
SYTL4	7.12E-07	3.58E+00
TCTA	7.18E-07	8.03E+00
CISH	7.18E-07	1.20E+01
SMAD1	7.29E-07	3.56E+00
LIG4	7.36E-07	4.61E+00
OARD1	7.76E-07	3.64E+00
NDUFV3	7.87E-07	2.90E+00
OSGEP	8.45E-07	4.68E+00
OPHN1	8.48E-07	4.76E+00
DTNBP1	8.56E-07	3.74E+00
TLR6	8.58E-07	6.01E+00
SLC16A14	8.62E-07	6.24E+00
HOXC11	8.66E-07	4.87E+00
EXPH5	8.85E-07	7.81E+00
PARD3B	9.42E-07	6.73E+00

DIXDC1	9.46E-07	3.94E+00
P2RY11	9.56E-07	5.68E+00
SLC4A8	9.70E-07	8.50E+00
AGA	1.02E-06	3.96E+00
PCBD2	1.03E-06	5.02E+00
KCTD18	1.06E-06	3.16E+00
SPAG16	1.07E-06	4.30E+00
ZNF75D	1.08E-06	4.95E+00
GAS8	1.08E-06	4.31E+00
ENSG00000231187	1.09E-06	3.90E+00
CCDC149	1.10E-06	7.98E+00
ERVK3-1	1.10E-06	2.89E+00
NTN1	1.10E-06	7.59E+00
ORMDL1	1.10E-06	3.91E+00
ALMS1	1.11E-06	2.77E+00
YJEFN3	1.11E-06	5.71E+00
SLC38A7	1.11E-06	3.61E+00
AMIGO1	1.12E-06	8.21E+00
THNSL2	1.14E-06	1.45E+01
PEX11A	1.18E-06	7.86E+00
ZNF346	1.18E-06	3.16E+00
MYB	1.21E-06	1.20E+01
MANSC1	1.21E-06	2.79E+00
CYP27A1	1.21E-06	6.27E+00
ZFAND2B	1.22E-06	3.41E+00
SDSL	1.23E-06	5.17E+00
POLH	1.26E-06	3.19E+00
C6orf47	1.28E-06	2.95E+00
MSL3P1	1.30E-06	5.51E+00
ZNF184	1.34E-06	4.41E+00
ZFAND2A	1.34E-06	6.76E+00
TTLL1	1.36E-06	7.15E+00
CARD8	1.37E-06	3.63E+00
ZSWIM7	1.38E-06	4.28E+00
CHMP4A	1.39E-06	2.77E+00
COX20	1.39E-06	5.81E+00
PPP1R3C	1.40E-06	6.13E+00
SIPA1L2	1.41E-06	5.93E+00
THTPA	1.41E-06	4.76E+00
ABHD11	1.43E-06	5.99E+00

MOSPD1	1.43E-06	4.09E+00
NME3	1.44E-06	4.68E+00
CDK5	1.45E-06	3.30E+00
SCNN1D	1.46E-06	6.17E+00
DENND2D	1.47E-06	3.94E+00
LMX1B	1.49E-06	4.49E+00
MPC1	1.49E-06	6.51E+00
C10orf95-AS1	1.51E-06	4.45E+00
TMEM18	1.59E-06	3.00E+00
TNFRSF11A	1.60E-06	5.19E+00
SRCIN1	1.61E-06	8.39E+00
SLC25A33	1.63E-06	4.13E+00
HMOX1	1.67E-06	4.59E+00
TRIM39	1.68E-06	2.71E+00
MRPL57	1.69E-06	2.45E+00
CYB5RL	1.72E-06	3.22E+00
EOLA1	1.73E-06	4.59E+00
SUGT1P4-STRA6LP-CCDC180	1.75E-06	5.49E+00
PPM1H	1.79E-06	3.86E+00
RNF152	1.84E-06	5.57E+00
PAFAH2	1.85E-06	3.70E+00
TCHP	1.86E-06	2.63E+00
TCEAL3	1.86E-06	3.30E+00
PGM2L1	1.87E-06	3.09E+00
TP53I3	1.88E-06	6.90E+00
PRADC1	1.88E-06	3.20E+00
ARHGAP26	1.89E-06	3.41E+00
ABHD5	1.91E-06	3.46E+00
RHPN1	1.92E-06	1.78E+01
BARD1	1.93E-06	2.92E+00
C1orf216	1.94E-06	4.68E+00
ABCD1	1.95E-06	6.51E+00
MC1R	1.95E-06	3.05E+00
HNMT	1.97E-06	9.98E+00
KIAA0895L	2.01E-06	3.65E+00
FHDC1	2.03E-06	5.21E+00
MUTYH	2.03E-06	4.02E+00
SEPTIN7P2	2.05E-06	3.58E+00
GRAMD1B	2.08E-06	5.55E+00
GUSBP11	2.10E-06	3.42E+00

ENTPD1	2.10E-06	5.76E+00
ZNF544	2.11E-06	2.49E+00
TRAPPC2L	2.11E-06	2.85E+00
CCL28	2.13E-06	2.59E+00
ATAD2B	2.15E-06	3.06E+00
MRPS11	2.16E-06	4.63E+00
CHDH	2.18E-06	1.28E+01
URB2	2.19E-06	2.52E+00
N6AMT1	2.19E-06	7.39E+00
FAM172A	2.22E-06	3.64E+00
OLMALINC	2.23E-06	2.71E+00
SIRT1	2.26E-06	2.92E+00
ADCK1	2.26E-06	3.36E+00
PANK1	2.29E-06	4.04E+00
GRTP1	2.32E-06	3.62E+00
CRISPLD2	2.34E-06	2.35E+00
USP18	2.39E-06	4.10E+00
F8	2.45E-06	4.91E+00
IBA57	2.46E-06	3.06E+00
PLCH1	2.47E-06	4.86E+00
PWWP2A	2.48E-06	2.51E+00
TNFRSF14	2.48E-06	5.94E+00
KIAA0895	2.48E-06	2.84E+00
MB	2.49E-06	1.70E+01
ATXN7L2	2.49E-06	4.66E+00
UBE2D4	2.49E-06	3.76E+00
MPHOSPH10	2.51E-06	3.18E+00
RNF113A	2.51E-06	2.76E+00
IFT27	2.56E-06	3.04E+00
PUSL1	2.56E-06	2.73E+00
ZBED3	2.57E-06	3.62E+00
BMP2K	2.60E-06	3.72E+00
MIS18A	2.62E-06	2.50E+00
CYB5D1	2.63E-06	3.59E+00
ILKAP	2.63E-06	2.74E+00
WWOX	2.63E-06	3.89E+00
TSPAN10	2.67E-06	1.03E+01
RAB20	2.68E-06	4.11E+00
FIG4	2.68E-06	2.27E+00
CASP8	2.68E-06	2.69E+00

EPB41L4A	2.70E-06	5.81E+00
FBF1	2.74E-06	2.63E+00
JADE1	2.74E-06	3.54E+00
PLPP5	2.75E-06	2.43E+00
MED17	2.78E-06	2.74E+00
SAP30	2.83E-06	4.28E+00
MSANTD2	2.85E-06	4.15E+00
PRR3	2.85E-06	2.93E+00
CCDC90B	2.88E-06	2.99E+00
PPAN-P2RY11	2.88E-06	7.19E+00
FPGT	2.95E-06	3.28E+00
CPPED1	2.97E-06	5.05E+00
GPR160	2.98E-06	2.66E+00
DNAAF2	3.00E-06	2.75E+00
SANBR	3.02E-06	3.86E+00
PARP3	3.04E-06	3.08E+00
UVRAG	3.05E-06	2.49E+00
ZNF524	3.08E-06	4.87E+00
RABEP2	3.12E-06	2.86E+00
NR1H3	3.14E-06	3.53E+00
SMIM3	3.15E-06	3.31E+00
IFT172	3.19E-06	3.37E+00
ZNF45	3.21E-06	3.09E+00
ADAL	3.27E-06	6.85E+00
DMXL1	3.31E-06	3.03E+00
LINC01224	3.31E-06	3.37E+00
RGL3	3.32E-06	2.95E+01
ZBTB18	3.32E-06	7.99E+00
ZNF579	3.33E-06	5.37E+00
VEPH1	3.35E-06	4.03E+00
FCGBP	3.36E-06	4.94E+00
MRNIP	3.44E-06	2.97E+00
FAM161B	3.46E-06	3.97E+00
RDH13	3.47E-06	2.43E+00
TMEM126B	3.49E-06	2.68E+00
HSD3B7	3.52E-06	6.00E+00
BFSP1	3.55E-06	6.16E+00
DNAJC30	3.56E-06	4.55E+00
ALG13	3.58E-06	2.68E+00
TMEM150A	3.60E-06	7.15E+00

RABL2B	3.63E-06	2.58E+00
EPC2	3.64E-06	2.31E+00
SRSF8	3.65E-06	3.71E+00
TMC5	3.66E-06	5.92E+00
LINC01128	3.67E-06	2.75E+00
ZNF776	3.67E-06	2.36E+00
KLF11	3.67E-06	9.69E+00
OVCA2	3.69E-06	2.55E+00
ENSG00000232098	3.74E-06	2.91E+00
RNF214	3.75E-06	2.68E+00
PCDHB9	3.75E-06	4.59E+00
IL17RE	3.83E-06	3.94E+00
ABCA12	3.83E-06	8.98E+00
CYB561D1	3.86E-06	2.42E+00
ZBTB8A	3.86E-06	2.81E+00
TCEAL1	3.87E-06	3.82E+00
NPAT	3.87E-06	2.45E+00
RGMB	3.92E-06	2.75E+00
PPARGC1B	3.93E-06	2.76E+00
PMS1	3.95E-06	3.40E+00
AADAT	3.95E-06	3.92E+00
SLFN13	3.98E-06	2.35E+00
RPP40	4.00E-06	4.46E+00
ZNF587	4.02E-06	2.27E+00
TRIM45	4.02E-06	5.18E+00
BCS1L	4.08E-06	3.29E+00
RABIF	4.09E-06	3.48E+00
CAPN7	4.10E-06	2.37E+00
KIAA1958	4.13E-06	3.03E+00
HEATR3	4.20E-06	2.75E+00
ENSG00000279207	4.26E-06	3.14E+00
ECHDC2	4.29E-06	2.21E+00
DUXAP8	4.30E-06	3.50E+00
CKMT1B	4.40E-06	4.15E+00
UBXN11	4.41E-06	3.40E+00
PDIK1L	4.48E-06	5.28E+00
LNP1	4.49E-06	4.57E+00
ZNF649	4.49E-06	6.24E+00
ZNF428	4.50E-06	3.46E+00
ENSG00000288884	4.50E-06	3.40E+00

GCAT	4.51E-06	5.45E+00
PRSS16	4.53E-06	2.14E+00
RIOK2	4.57E-06	4.06E+00
ATG4A	4.63E-06	4.29E+00
GLIDR	4.64E-06	4.05E+00
XPNPEP3	4.68E-06	2.31E+00
SLC25A4	4.68E-06	2.41E+00
BPHL	4.69E-06	2.72E+00
CCNQ	4.85E-06	2.46E+00
CMTM8	4.90E-06	3.71E+00
TRPM2	4.91E-06	3.37E+00
FMC1	4.91E-06	4.85E+00
ENSG00000272512	4.93E-06	4.33E+00
POP5	4.98E-06	3.46E+00
PHTF1	4.98E-06	2.85E+00
NHEJ1	4.98E-06	3.97E+00
TIRAP	5.06E-06	3.90E+00
IL1R1	5.07E-06	2.94E+00
ZNF329	5.09E-06	2.65E+00
ST7L	5.14E-06	3.54E+00
ZW10	5.15E-06	2.89E+00
WDR7	5.17E-06	2.76E+00
PASK	5.17E-06	2.44E+00
ENSG00000274173	5.17E-06	4.19E+00
SLC29A2	5.17E-06	4.08E+00
PRXL2B	5.19E-06	5.59E+00
ASNSD1	5.29E-06	3.21E+00
PMVK	5.30E-06	2.79E+00
DNAJC22	5.30E-06	1.01E+01
ZNF354A	5.32E-06	3.06E+00
ACOT13	5.42E-06	2.33E+00
AARS2	5.47E-06	2.26E+00
TPMT	5.69E-06	2.13E+00
TTC38	5.69E-06	2.94E+00
CAPN8	5.72E-06	5.92E+00
LAMA4	5.72E-06	5.07E+00
CDKN2D	5.75E-06	2.37E+00
CUTALP	5.77E-06	3.53E+00
SLC25A19	5.92E-06	3.00E+00
TMEM50B	6.05E-06	2.42E+00

CAB39L	6.05E-06	3.31E+00
BARX2	6.05E-06	3.16E+00
LINC01133	6.06E-06	4.28E+00
MTARC2	6.06E-06	2.63E+00
VPS9D1	6.13E-06	2.53E+00
YJU2B	6.13E-06	2.41E+00
CDC7	6.15E-06	2.47E+00
TATDN3	6.16E-06	5.00E+00
ZNF750	6.29E-06	4.90E+00
AFG3L1P	6.32E-06	3.29E+00
EIF2B4	6.33E-06	2.20E+00
CKMT1A	6.34E-06	3.28E+00
CADM4	6.36E-06	4.49E+00
METTL8	6.40E-06	2.72E+00
ZNF827	6.41E-06	2.39E+00
ARRDC3	6.45E-06	3.91E+00
RANBP10	6.45E-06	2.28E+00
INTS4	6.48E-06	2.29E+00
HFE	6.52E-06	2.72E+00
OSER1-DT	6.53E-06	4.94E+00
MRM3	6.54E-06	2.55E+00
ICAM3	6.54E-06	3.99E+00
BBOF1	6.63E-06	5.49E+00
ATP2C2	6.63E-06	7.69E+00
C1orf210	6.70E-06	5.91E+00
GET1	6.84E-06	2.33E+00
GON7	6.99E-06	3.69E+00
IQGAP2	7.01E-06	7.71E+00
TEN1	7.01E-06	2.81E+00
EGFL7	7.01E-06	8.81E+00
ZNF117	7.03E-06	3.77E+00
DOLPP1	7.08E-06	2.24E+00
ARPIN-AP3S2	7.10E-06	2.66E+00
EPC1	7.15E-06	3.52E+00
PGAP1	7.17E-06	2.77E+00
HDAC4	7.21E-06	2.97E+00
SMIM12	7.30E-06	2.23E+00
ZNF627	7.30E-06	4.89E+00
TSHZ1	7.30E-06	2.80E+00
LINC00662	7.32E-06	2.41E+00

CASP10	7.36E-06	3.83E+00
STRA6	7.38E-06	2.74E+00
MACO1	7.42E-06	2.37E+00
SLC4A11	7.50E-06	2.21E+00
C1orf50	7.52E-06	3.25E+00
SLC13A3	7.54E-06	4.23E+00
PNKP	7.66E-06	2.24E+00
RBMXL1	7.72E-06	3.03E+00
SP4	7.74E-06	2.52E+00
ALG6	7.76E-06	3.28E+00
CFD	7.78E-06	1.11E+01
TFPT	7.81E-06	2.85E+00
PCBP1-AS1	7.89E-06	6.45E+00
B4GALNT3	7.93E-06	2.64E+00
ALKBH2	7.98E-06	2.89E+00
ZNF276	8.08E-06	2.14E+00
ZNF74	8.09E-06	2.70E+00
KIF27	8.11E-06	3.78E+00
RIMS3	8.12E-06	2.60E+00
CRYBB2P1	8.13E-06	3.19E+00
SBDSP1	8.37E-06	3.21E+00
DOK3	8.51E-06	3.71E+00
KLHL18	8.55E-06	2.40E+00
TMEM254	8.66E-06	3.79E+00
TIMM21	8.71E-06	2.68E+00
CROCCP2	8.72E-06	2.30E+00
RHBDD3	8.74E-06	2.30E+00
GTF2IP13	8.82E-06	2.98E+00
PRR19	8.85E-06	4.89E+00
PGGT1B	8.91E-06	3.53E+00
KYAT3	8.91E-06	2.33E+00
TLR5	8.92E-06	5.89E+00
PLSCR4	9.03E-06	2.47E+00
ANKMY1	9.04E-06	3.40E+00
KLHL22	9.07E-06	2.02E+00
AK9	9.14E-06	3.46E+00
NBPF12	9.22E-06	2.09E+00
DUS3L	9.22E-06	2.74E+00
NUDT12	9.26E-06	3.72E+00
STK16	9.27E-06	2.09E+00

PLCE1	9.31E-06	3.11E+00
RBBP8NL	9.36E-06	6.62E+00
ERVK13-1	9.37E-06	3.22E+00
GCHFR	9.39E-06	4.69E+00
YY2	9.44E-06	4.22E+00
TMEM218	9.44E-06	3.93E+00
DNAJC6	9.53E-06	4.43E+00
POLI	9.64E-06	2.42E+00
RNF14	9.69E-06	2.25E+00
GBAP1	9.70E-06	6.21E+00
ASB16-AS1	9.70E-06	3.06E+00
AEN	9.79E-06	2.80E+00
FTX	9.84E-06	2.43E+00
TSPYL5	9.87E-06	5.88E+00
LRIG2	9.89E-06	2.15E+00
NRAV	9.92E-06	4.40E+00
PAQR6	1.01E-05	4.26E+00
ALS2	1.01E-05	2.25E+00
ZNF417	1.03E-05	2.40E+00
ARV1	1.03E-05	2.46E+00
RNF25	1.04E-05	3.17E+00
LRATD1	1.05E-05	7.59E+00
CICP14	1.05E-05	3.08E+00
IGFBP2	1.05E-05	3.62E+00
ZNF606	1.05E-05	2.71E+00
ELP6	1.06E-05	3.39E+00
PABIR1	1.06E-05	2.51E+00
HYAL3	1.06E-05	2.54E+00
NIPSNAP3A	1.07E-05	2.58E+00
SLC40A1	1.07E-05	8.20E+00
MGST2	1.08E-05	2.51E+00
UMAD1	1.08E-05	2.36E+00
C2orf15	1.09E-05	3.88E+00
SLC25A20	1.09E-05	3.68E+00
IFT46	1.09E-05	3.94E+00
CBX8	1.09E-05	3.69E+00
ZNF497	1.11E-05	5.60E+00
AP4B1	1.11E-05	2.76E+00
FAM126B	1.12E-05	3.54E+00
ENTPD1-AS1	1.12E-05	4.28E+00

RNF115	1.13E-05	2.20E+00
PCCA-DT	1.15E-05	3.37E+00
AP4M1	1.15E-05	2.73E+00
DTX2P1-UPK3BP1-PMS2P11	1.17E-05	3.15E+00
EEF2KMT	1.18E-05	2.38E+00
TDRKH	1.18E-05	3.11E+00
PSCA	1.19E-05	3.94E+00
TBCEL	1.19E-05	2.95E+00
ENPP4	1.19E-05	2.58E+00
ZNF529	1.19E-05	2.76E+00
ANKRD54	1.20E-05	2.06E+00
INO80D	1.20E-05	3.35E+00
FANCE	1.21E-05	2.43E+00
ETFDH	1.21E-05	2.40E+00
AAMDC	1.23E-05	3.89E+00
FEM1A	1.24E-05	2.53E+00
BUD13	1.24E-05	2.01E+00
SAYSD1	1.25E-05	4.21E+00
ARHGAP31	1.25E-05	2.35E+00
LSM10	1.25E-05	2.20E+00
GAB1	1.27E-05	2.78E+00
DOCK8	1.29E-05	7.11E+00
GFOD2	1.29E-05	2.03E+00
ORC1	1.30E-05	2.23E+00
CDRT1	1.30E-05	5.35E+00
AZI2	1.31E-05	2.08E+00
TMTC4	1.31E-05	2.66E+00
FBXL17	1.31E-05	2.90E+00
BEND3	1.32E-05	2.57E+00
ZNF837	1.32E-05	8.44E+00
ACYP2	1.33E-05	3.66E+00
FNIP2	1.36E-05	2.66E+00
ZNF449	1.36E-05	2.91E+00
EXTL2	1.37E-05	2.97E+00
MCRIP2	1.38E-05	2.18E+00
GSDMC	1.39E-05	2.47E+00
IQANK1	1.39E-05	3.27E+00
ELF2	1.39E-05	2.08E+00
ANKMY2	1.39E-05	2.44E+00
ZFP1	1.39E-05	3.09E+00

H2BC5	1.40E-05	1.04E+01
BICRAL	1.41E-05	2.04E+00
DHRS4	1.44E-05	2.91E+00
C7orf26	1.44E-05	2.26E+00
GRHL3	1.48E-05	3.20E+00
GABPB2	1.48E-05	4.23E+00
ZGLP1	1.52E-05	7.01E+00
RBAK	1.53E-05	2.39E+00
SLC12A2-DT	1.56E-05	2.10E+00
SLC25A35	1.56E-05	4.22E+00
PGBD2	1.60E-05	3.67E+00
GALNT11	1.60E-05	2.11E+00
DPY19L3	1.60E-05	2.58E+00
PRR15	1.60E-05	3.10E+00
AMDHD2	1.60E-05	2.39E+00
PCSK1N	1.62E-05	8.97E+00
OGG1	1.62E-05	2.83E+00
ZNF12	1.63E-05	2.07E+00
ZNF443	1.68E-05	5.91E+00
REEP1	1.69E-05	9.84E+00
CSAD	1.69E-05	3.46E+00
ZNF440	1.73E-05	2.28E+00
ME3	1.73E-05	4.29E+00
TCEANC2	1.73E-05	2.23E+00
ENSG00000260121	1.74E-05	4.05E+00
EME2	1.74E-05	2.20E+00
H2BC20P	1.74E-05	3.98E+00
SIAH1	1.75E-05	2.01E+00
ZBTB14	1.75E-05	2.13E+00
GTF2IRD2B	1.75E-05	3.12E+00
ABHD14B	1.76E-05	2.30E+00
KCNK5	1.76E-05	2.34E+00
SCAMP1-AS1	1.77E-05	4.51E+00
USP38	1.79E-05	2.38E+00
CRTC1	1.81E-05	2.80E+00
MLH1	1.81E-05	2.41E+00
ELMOD3	1.81E-05	2.02E+00
PGAP3	1.84E-05	2.22E+00
ZIC5	1.87E-05	4.85E+00
ZNF354C	1.89E-05	2.57E+00

NUDT8	1.90E-05	4.96E+00
COA5	1.90E-05	2.40E+00
MED26	1.90E-05	2.36E+00
WNK4	1.93E-05	4.15E+00
MAD1L1	1.93E-05	2.22E+00
ZNF433	1.93E-05	3.38E+00
CCNE1	1.98E-05	3.85E+00
CCNH	1.98E-05	2.53E+00
ZBED6CL	2.02E-05	2.68E+00
CTSF	2.02E-05	1.00E+01
BORCS7	2.04E-05	2.46E+00
ENSG00000288849	2.04E-05	3.92E+00
HSD17B2	2.04E-05	9.63E+00
TBC1D3L	2.05E-05	2.72E+00
ACACB	2.05E-05	3.00E+00
MINDY4	2.05E-05	3.47E+00
CTSO	2.05E-05	5.34E+00
TEN1-CDK3	2.06E-05	2.34E+00
SRP19	2.06E-05	2.97E+00
LRRC3	2.07E-05	8.19E+00
FAM114A2	2.07E-05	2.56E+00
TRAPPC13	2.08E-05	2.64E+00
GCFC2	2.09E-05	2.30E+00
LYSMD3	2.11E-05	3.55E+00
COMTD1	2.11E-05	3.22E+00
PIGM	2.11E-05	3.46E+00
KCTD15	2.12E-05	4.42E+00
RP9P	2.12E-05	2.85E+00
ARRDC2	2.13E-05	2.72E+00
CRACD	2.16E-05	2.78E+00
TMEM198B	2.17E-05	2.86E+00
NSUN4	2.17E-05	2.26E+00
TNRC6C	2.19E-05	2.98E+00
FXN	2.19E-05	2.88E+00
TUBA5P	2.19E-05	7.95E+00
EP400P1	2.25E-05	3.69E+00
SGF29	2.26E-05	2.72E+00
BDH2	2.27E-05	2.96E+00
CTC1	2.29E-05	2.65E+00
ENSG00000262160	2.29E-05	2.35E+00

FASTKD1	2.29E-05	2.40E+00
STMN3	2.29E-05	3.33E+00
EXOC6	2.29E-05	2.68E+00
TCN2	2.30E-05	3.45E+00
COQ2	2.31E-05	2.01E+00
SPATA33	2.32E-05	3.53E+00
CTU2	2.32E-05	2.26E+00
ZNF682	2.34E-05	3.63E+00
PSMA3-AS1	2.34E-05	2.62E+00
FAM185A	2.35E-05	3.09E+00
ZNF875	2.35E-05	2.14E+00
PPIAP22	2.35E-05	2.03E+00
CNOT10	2.39E-05	2.11E+00
DXO	2.39E-05	2.11E+00
CSTF3	2.40E-05	2.13E+00
ZNF700	2.42E-05	2.48E+00
ZNF846	2.43E-05	4.79E+00
WHRN	2.48E-05	3.82E+00
ENSG00000285589	2.49E-05	2.78E+00
AUNIP	2.53E-05	2.32E+00
AMACR	2.55E-05	3.18E+00
RNF121	2.55E-05	2.23E+00
GINS2	2.57E-05	2.28E+00
SETMAR	2.59E-05	7.90E+00
SPINT1-AS1	2.59E-05	3.38E+00
ZNF44	2.62E-05	3.71E+00
CCDC15	2.62E-05	3.92E+00
GNAO1	2.63E-05	2.89E+00
ANKHD1-EIF4EBP3	2.64E-05	2.88E+00
ENSG00000229689	2.64E-05	3.84E+00
SALL2	2.65E-05	6.16E+00
MAMDC4	2.66E-05	4.19E+00
CLTCL1	2.66E-05	2.87E+00
NHSL1	2.68E-05	2.30E+00
ZNF407	2.70E-05	2.58E+00
GAREM1	2.71E-05	3.23E+00
MZF1	2.73E-05	2.68E+00
SNHG15	2.74E-05	3.14E+00
ZNF607	2.74E-05	2.10E+00
ANGEL2	2.75E-05	2.07E+00

ZNF260	2.78E-05	2.63E+00
ZNF302	2.82E-05	2.97E+00
NGEF	2.82E-05	3.75E+00
NUP42	2.83E-05	2.37E+00
PI4KAP2	2.90E-05	2.14E+00
H2BC19P	2.90E-05	4.67E+00
RHOU	2.91E-05	4.73E+00
NT5C3A	2.94E-05	2.36E+00
PDXP	2.96E-05	2.17E+00
ZNF737	2.97E-05	3.91E+00
NEU3	2.99E-05	2.68E+00
KCTD21	3.00E-05	2.58E+00
YBEY	3.04E-05	4.18E+00
ENSG00000212978	3.06E-05	3.24E+00
APBB3	3.07E-05	2.20E+00
SUOX	3.07E-05	2.69E+00
PRSS27	3.10E-05	5.13E+00
ENSG00000273151	3.10E-05	4.08E+00
CLYBL	3.12E-05	3.31E+00
ILDR1	3.18E-05	2.88E+00
RMI1	3.18E-05	2.22E+00
TAF13	3.24E-05	2.81E+00
ENSG00000277117	3.25E-05	3.79E+00
HOMER1	3.25E-05	2.03E+00
LINC00667	3.29E-05	2.01E+00
CPE	3.30E-05	3.06E+00
PSMC3IP	3.30E-05	2.95E+00
AKTIP	3.31E-05	2.11E+00
NEK11	3.32E-05	3.42E+00
TMLHE	3.32E-05	3.01E+00
ZNF786	3.33E-05	2.17E+00
RGL1	3.36E-05	3.88E+00
SURF2	3.39E-05	2.55E+00
HCG18	3.39E-05	2.04E+00
UGT1A6	3.40E-05	7.32E+00
CFAP298-TCP10L	3.40E-05	5.44E+00
SLC39A4	3.44E-05	6.17E+00
OR7E14P	3.47E-05	4.80E+00
ZNF138	3.48E-05	3.04E+00
MYLIP	3.49E-05	2.57E+00

TMED7-TICAM2	3.50E-05	2.38E+00
STAG3L3	3.50E-05	2.92E+00
PUS3	3.50E-05	4.14E+00
KANSL1L	3.53E-05	3.41E+00
TENT5B	3.59E-05	4.89E+00
RIBC2	3.59E-05	4.69E+00
ZNF202	3.64E-05	2.20E+00
FAM225A	3.66E-05	4.23E+00
TEX30	3.70E-05	2.55E+00
C7orf25	3.71E-05	3.41E+00
ZNF552	3.75E-05	3.25E+00
ATP9B	3.77E-05	2.68E+00
RRAGB	3.78E-05	3.19E+00
SYNM	3.79E-05	3.74E+00
B3GALNT1	3.81E-05	2.64E+00
TMBIM4	3.81E-05	2.12E+00
RAB19	3.81E-05	3.29E+00
CARF	3.83E-05	4.48E+00
USP32P3	3.85E-05	2.83E+00
ENSG00000176593	3.86E-05	2.41E+00
ZNF814	3.88E-05	2.42E+00
PEX7	3.89E-05	2.93E+00
MON1A	3.91E-05	2.36E+00
PTMAP5	3.91E-05	2.51E+00
RIPK1	3.91E-05	2.13E+00
DYNC2LI1	3.91E-05	2.63E+00
ENSG00000274276	3.92E-05	7.86E+00
C2orf69	3.93E-05	2.71E+00
ZNF844	3.93E-05	2.44E+00
DHRS4L2	3.95E-05	2.72E+00
RNASEH1-DT	3.97E-05	3.39E+00
THEM4	3.97E-05	2.14E+00
RPP25	4.01E-05	2.40E+00
TXNDC16	4.01E-05	2.06E+00
ZNF791	4.05E-05	2.09E+00
ABCB6	4.05E-05	2.09E+00
RUFY3	4.06E-05	2.12E+00
MPV17L2	4.07E-05	2.12E+00
SPC25	4.08E-05	3.36E+00
NINL	4.10E-05	2.91E+00

LINC01963	4.13E-05	2.89E+00
SFXN2	4.16E-05	3.09E+00
HSF4	4.16E-05	2.28E+00
MAIP1	4.16E-05	2.32E+00
CLK4	4.16E-05	2.80E+00
UHRF1BP1L	4.17E-05	2.08E+00
SLC18B1	4.17E-05	2.13E+00
METTL4	4.21E-05	2.38E+00
CFAP119	4.35E-05	3.45E+00
MIR924HG	4.35E-05	3.58E+00
ZNF836	4.38E-05	2.57E+00
BACE1-AS	4.44E-05	3.50E+00
TM7SF2	4.45E-05	2.09E+00
EPM2AIP1	4.45E-05	2.95E+00
C11orf98	4.45E-05	2.03E+00
COQ8A	4.47E-05	2.21E+00
BTBD9	4.49E-05	2.53E+00
SEPTIN7P14	4.52E-05	3.83E+00
ZNHIT2	4.53E-05	2.17E+00
MARS2	4.53E-05	2.95E+00
ENSG00000269825	4.53E-05	2.71E+00
FKBP5	4.56E-05	2.05E+00
ZNF599	4.58E-05	3.02E+00
GGACT	4.69E-05	5.97E+00
GLCCI1	4.74E-05	2.63E+00
PHYKPL	4.80E-05	2.11E+00
ZFP30	4.82E-05	2.16E+00
PTS	4.82E-05	2.12E+00
RSAD1	4.83E-05	2.13E+00
ZNF181	4.83E-05	3.77E+00
ACTR3B	4.86E-05	2.26E+00
ZNF589	4.86E-05	3.22E+00
IFT22	4.86E-05	2.22E+00
WIPI1	4.86E-05	2.84E+00
EED	4.87E-05	2.02E+00
PGPEP1	4.87E-05	2.46E+00
ZNF436	4.87E-05	2.29E+00
DUXAP9	4.94E-05	3.01E+00
MIOS	4.96E-05	2.01E+00
CWF19L2	5.12E-05	2.59E+00

ZNF586	5.14E-05	2.59E+00
NFKBIL1	5.16E-05	2.59E+00
REXO5	5.20E-05	2.53E+00
PIAS2	5.22E-05	2.02E+00
KRT8P12	5.28E-05	3.20E+00
ZNF605	5.31E-05	2.27E+00
ZNF519	5.36E-05	2.42E+00
APTR	5.37E-05	2.87E+00
MCAT	5.38E-05	2.78E+00
TOE1	5.43E-05	4.17E+00
ADIRF-AS1	5.51E-05	2.98E+00
EFNA3	5.55E-05	4.65E+00
HEXIM2	5.55E-05	3.42E+00
ZSCAN31	5.58E-05	4.69E+00
TIMM29	5.64E-05	2.45E+00
ZNF493	5.64E-05	2.90E+00
RNF227	5.64E-05	3.63E+00
SCML2	5.66E-05	2.28E+00
ZNF512	5.68E-05	2.23E+00
SEC11C	5.74E-05	3.19E+00
NDUFA6-DT	5.75E-05	3.03E+00
ZNF616	5.79E-05	2.44E+00
ZNF593	5.81E-05	2.24E+00
ZNF471	5.83E-05	2.83E+00
TIMM10B	5.83E-05	2.17E+00
RP9	5.83E-05	2.16E+00
DCAF17	5.84E-05	2.10E+00
SLC1A1	5.87E-05	2.60E+00
CCDC34	5.87E-05	2.21E+00
PPARG	6.04E-05	2.60E+00
DYRK1B	6.05E-05	2.83E+00
NEMP2	6.20E-05	2.57E+00
ZNF14	6.21E-05	2.41E+00
ZNF558	6.25E-05	2.32E+00
LDLRAD3	6.35E-05	2.37E+00
ZNF204P	6.41E-05	2.40E+00
CCDC125	6.41E-05	2.82E+00
MMACHC	6.42E-05	2.88E+00
P4HTM	6.42E-05	2.54E+00
ZFAND2A-DT	6.43E-05	3.13E+00

TMEM251	6.43E-05	2.40E+00
MIR1915HG	6.45E-05	2.28E+00
CBR4	6.46E-05	3.09E+00
GTF3C6	6.51E-05	2.23E+00
LINC00467	6.56E-05	2.73E+00
GDAP2	6.57E-05	2.06E+00
SSBP2	6.57E-05	2.93E+00
ACP3	6.61E-05	3.47E+00
RBMX2	6.65E-05	2.09E+00
AJM1	6.67E-05	3.78E+00
FGGY	6.71E-05	2.88E+00
ZNF653	6.73E-05	2.70E+00
ATPSCKMT	6.75E-05	2.26E+00
U2AF1L4	6.78E-05	3.19E+00
SWI5	6.80E-05	2.13E+00
HLA-H	6.82E-05	2.99E+00
TIMM8A	6.84E-05	2.36E+00
PCLO	6.84E-05	2.95E+00
FAM151B-DT	6.84E-05	2.33E+00
B3GALT4	6.85E-05	3.67E+00
TMEM238	6.94E-05	3.43E+00
VRK2	6.95E-05	2.08E+00
IPP	7.03E-05	2.26E+00
ENSG00000257337	7.06E-05	3.33E+00
HOXA5	7.10E-05	2.48E+00
NOL3	7.13E-05	2.46E+00
ACSF3	7.13E-05	2.26E+00
SIK3	7.14E-05	2.04E+00
SNRNP48	7.23E-05	2.05E+00
ENSG00000273015	7.43E-05	3.79E+00
C19orf44	7.44E-05	2.66E+00
AGO3	7.48E-05	2.05E+00
TYMSOS	7.48E-05	5.80E+00
MBD5	7.52E-05	2.03E+00
ZNF480	7.53E-05	2.09E+00
MICU2	7.59E-05	2.19E+00
ENSG00000230882	7.63E-05	1.24E+01
ZNF397	7.67E-05	2.70E+00
RYR1	7.69E-05	2.78E+00
HGH1	7.70E-05	2.18E+00

ENTPD2	7.73E-05	4.42E+00
HAGHL	7.78E-05	2.19E+00
DOP1A	7.79E-05	2.01E+00
TIGAR	7.82E-05	3.31E+00
ENSG00000269604	7.82E-05	2.03E+00
ZNF91	7.93E-05	2.22E+00
SLC6A17	7.95E-05	8.98E+00
KBTBD4	8.05E-05	2.09E+00
ZNF771	8.17E-05	4.57E+00
PLEKHA8P1	8.24E-05	3.10E+00
CSRNP1	8.25E-05	2.05E+00
SNHG20	8.29E-05	3.22E+00
MRPL22	8.29E-05	2.00E+00
FAAP24	8.29E-05	3.29E+00
ZNF429	8.32E-05	2.09E+00
ACTR8	8.33E-05	2.11E+00
CAMKMT	8.36E-05	3.42E+00
NDUFA1	8.59E-05	2.72E+00
CSAG3	8.62E-05	3.30E+00
ZSCAN9	8.67E-05	2.18E+00
SNX21	8.70E-05	2.50E+00
SLC35B3	8.70E-05	2.03E+00
SPRTN	8.73E-05	2.21E+00
PPP1R3E	8.77E-05	2.29E+00
BBS1	8.81E-05	2.05E+00
TCAF1P1	8.85E-05	2.03E+00
DNAH10OS	8.88E-05	3.64E+00
CDK20	8.97E-05	2.49E+00
ZBTB42	9.00E-05	2.69E+00
SAMD10	9.03E-05	2.12E+00
RAB40B	9.24E-05	2.44E+00
FBXO4	9.25E-05	2.08E+00
POLR3C	9.29E-05	2.10E+00
GRB14	9.34E-05	4.24E+00
ANKRD42	9.46E-05	2.17E+00
ZNF816	9.47E-05	2.35E+00
ZNF490	9.51E-05	3.42E+00
CTSK	9.56E-05	3.45E+00
CTNS	9.59E-05	2.02E+00
ZNF615	9.69E-05	2.18E+00

TRAF5	9.71E-05	2.00E+00
ZNF124	9.74E-05	3.48E+00
NFATC2	9.84E-05	4.15E+00
DHRS11	9.90E-05	2.05E+00
RBM34	9.99E-05	2.15E+00
ALKBH7	1.01E-04	2.90E+00
METTL21A	1.01E-04	2.04E+00
ARHGAP24	1.02E-04	3.35E+00
THOC5	1.02E-04	2.42E+00
ENSG00000254837	1.03E-04	3.59E+00
LMF1	1.03E-04	3.04E+00
KLHL26	1.03E-04	2.29E+00
ZNF785	1.07E-04	2.28E+00
RNF216P1	1.08E-04	2.01E+00
STRADB	1.09E-04	2.07E+00
CDON	1.10E-04	3.30E+00
RASA3	1.11E-04	2.29E+00
PAXIP1-DT	1.11E-04	2.36E+00
USP35	1.11E-04	2.14E+00
ZNF234	1.11E-04	2.15E+00
ZNF280C	1.12E-04	3.07E+00
SLC22A20P	1.13E-04	3.27E+00
ENSG00000280441	1.13E-04	2.20E+00
PCSK6	1.14E-04	2.23E+00
EDN2	1.14E-04	6.38E+00
ANTKMT	1.15E-04	2.24E+00
ENSG00000228463	1.15E-04	6.15E+00
ZNF69	1.16E-04	2.53E+00
CTC-338M12.4	1.17E-04	2.79E+00
MTRES1	1.17E-04	2.84E+00
FAM241B	1.17E-04	2.15E+00
BTD	1.17E-04	2.16E+00
ZFP69	1.17E-04	3.17E+00
KCTD7	1.18E-04	2.55E+00
RPGRIP1L	1.19E-04	2.42E+00
ZNF784	1.19E-04	3.16E+00
MFSD8	1.19E-04	2.16E+00
IPO5P1	1.21E-04	2.83E+00
ZNF33B	1.24E-04	2.79E+00
APOLD1	1.25E-04	2.22E+00

ARMCX5	1.25E-04	2.33E+00
EXO5	1.26E-04	2.32E+00
BBS4	1.27E-04	2.12E+00
COLCA2	1.27E-04	3.53E+00
ABHD13	1.27E-04	2.37E+00
CCDC157	1.28E-04	4.13E+00
SIAE	1.28E-04	2.45E+00
MPP3	1.29E-04	2.41E+00
CCNE2	1.30E-04	3.17E+00
PARD6A	1.30E-04	5.52E+00
STARD7-AS1	1.32E-04	2.93E+00
LINC01547	1.34E-04	2.54E+00
TRMO	1.35E-04	2.85E+00
APIP	1.35E-04	2.10E+00
ZNF774	1.35E-04	3.35E+00
RTN4RL2	1.36E-04	3.85E+00
TRIM2	1.37E-04	2.10E+00
ZNF415	1.38E-04	2.15E+00
FAM120C	1.40E-04	2.06E+00
LRRC51	1.41E-04	2.95E+00
CENPS	1.43E-04	2.27E+00
DUSP23	1.44E-04	2.08E+00
UBA6-DT	1.48E-04	2.31E+00
TTC28-AS1	1.48E-04	2.22E+00
ENSG00000280128	1.48E-04	2.63E+00
LINC00265	1.49E-04	2.19E+00
MVB12B	1.49E-04	2.35E+00
SORBS2	1.50E-04	2.78E+00
METAP1D	1.51E-04	2.58E+00
ENSG00000288953	1.51E-04	2.79E+00
STK32C	1.52E-04	2.44E+00
MED7	1.52E-04	2.68E+00
ZNF691	1.52E-04	2.11E+00
PBLD	1.52E-04	3.30E+00
ENSG00000277758	1.63E-04	2.62E+00
TUSC1	1.63E-04	5.81E+00
SMCO4	1.64E-04	5.02E+00
TRIM52	1.65E-04	2.53E+00
CENATAC	1.65E-04	2.10E+00
GPR137C	1.67E-04	3.01E+00

RAPGEF4	1.70E-04	2.45E+00
ARMC7	1.70E-04	2.05E+00
ZNF789	1.70E-04	2.73E+00
FBXO43	1.71E-04	2.85E+00
ABCF2-H2BE1	1.71E-04	2.61E+00
ZNF232	1.75E-04	2.31E+00
ZNF354B	1.78E-04	3.06E+00
NUDT4B	1.78E-04	2.68E+00
THNSL1	1.80E-04	2.50E+00
HSD11B1L	1.82E-04	2.84E+00
AKR1B15	1.83E-04	2.42E+00
NPIPB15	1.85E-04	3.14E+00
ZNF35	1.86E-04	2.96E+00
MRPL39	1.86E-04	2.32E+00
ZNF283	1.86E-04	2.38E+00
ZNF684	1.87E-04	3.12E+00
LINC00680	1.90E-04	3.44E+00
ENSG00000289047	1.93E-04	2.20E+00
CNPY4	1.95E-04	2.01E+00
POGLUT1	1.96E-04	2.17E+00
ZNF568	1.96E-04	3.02E+00
EPOR	1.96E-04	2.64E+00
FBXO31	1.97E-04	2.08E+00
WNK3	1.98E-04	2.71E+00
ANAPC1P2	1.99E-04	2.24E+00
RGPD1	1.99E-04	2.57E+00
TTC32	1.99E-04	3.00E+00
ZNF18	2.01E-04	2.74E+00
SIL1	2.02E-04	2.01E+00
ZNF611	2.03E-04	2.19E+00
HIBCH	2.03E-04	2.49E+00
TMEM86A	2.04E-04	4.90E+00
CCDC126	2.08E-04	2.66E+00
DLX3	2.11E-04	2.52E+00
ENSG00000227766	2.11E-04	2.97E+00
ZNF66	2.12E-04	3.04E+00
ZNF805	2.12E-04	2.43E+00
ZNF398	2.13E-04	2.03E+00
FAM241A	2.15E-04	2.04E+00
PTCH1	2.15E-04	2.01E+00

MBLAC2	2.15E-04	2.24E+00
CRYZL2P	2.17E-04	2.05E+00
LINC01003	2.17E-04	3.11E+00
MRPS17	2.19E-04	2.04E+00
ZMPSTE24-DT	2.20E-04	4.80E+00
FAM98C	2.22E-04	2.28E+00
GLMN	2.24E-04	2.29E+00
TRIM62	2.28E-04	2.17E+00
ZNF17	2.29E-04	2.18E+00
CENPBD1P	2.30E-04	2.19E+00
MYL5	2.30E-04	3.07E+00
SPATA2L	2.30E-04	2.42E+00
THAP8	2.31E-04	2.45E+00
MLYCD	2.32E-04	3.41E+00
KHK	2.36E-04	2.23E+00
ZNF577	2.40E-04	2.91E+00
ZNF726	2.42E-04	2.83E+00
ZNF93	2.43E-04	2.03E+00
TMEM161B-DT	2.43E-04	2.14E+00
SERTAD1	2.47E-04	2.40E+00
RBM45	2.48E-04	2.34E+00
HECTD2	2.49E-04	2.62E+00
ARSA	2.53E-04	2.04E+00
EFNA4	2.59E-04	2.26E+00
LIPT2	2.62E-04	2.64E+00
KCNIP3	2.63E-04	2.78E+00
ZNF333	2.64E-04	2.43E+00
GATB	2.64E-04	2.34E+00
SLC31A2	2.68E-04	3.13E+00
C11orf1	2.77E-04	2.31E+00
ROCK1P1	2.82E-04	2.88E+00
ZNF70	2.85E-04	2.35E+00
FGF1	2.90E-04	4.35E+00
RNF223	2.92E-04	9.26E+00
HABP4	2.94E-04	2.24E+00
FAM13A	2.94E-04	2.15E+00
LRRC37A3	2.96E-04	3.15E+00
TMEM116	2.96E-04	2.71E+00
GAS2L3	2.96E-04	2.16E+00
TMEM126A	3.00E-04	2.07E+00

PCED1B	3.02E-04	2.32E+00
COMMD1	3.03E-04	2.03E+00
POLR2J4	3.11E-04	2.35E+00
CLBA1	3.12E-04	2.02E+00
TMEM191A	3.16E-04	2.29E+00
CASTOR2	3.16E-04	2.14E+00
TCHH	3.19E-04	2.51E+00
CHAC2	3.28E-04	3.89E+00
SPRY1	3.30E-04	2.69E+00
CCDC112	3.30E-04	2.36E+00
CBR3	3.32E-04	2.02E+00
CD27-AS1	3.47E-04	2.49E+00
C4A	3.47E-04	6.68E+00
PECR	3.50E-04	2.10E+00
TSPOAP1	3.52E-04	2.50E+00
TIGD7	3.53E-04	2.23E+00
PNPLA3	3.55E-04	3.58E+00
AGAP6	3.55E-04	2.87E+00
OXNAD1	3.56E-04	2.28E+00
ENSG00000261526	3.56E-04	2.70E+00
ZNF564	3.58E-04	3.11E+00
ENSG00000237807	3.61E-04	3.66E+00
FBXL15	3.62E-04	2.10E+00
ENSG00000260267	3.65E-04	2.54E+00
MORN2	3.73E-04	2.08E+00
TSHZ2	3.77E-04	2.32E+00
ZNF439	3.78E-04	2.54E+00
PRKAA2	3.87E-04	2.00E+00
ABHD18	3.90E-04	2.22E+00
PDPK2P	3.97E-04	2.39E+00
C1orf56	3.99E-04	2.43E+00
ASAH2B	4.02E-04	2.31E+00
ZNF566	4.14E-04	2.39E+00
WDR5B	4.14E-04	2.22E+00
MAGI1	4.16E-04	2.22E+00
SHROOM1	4.17E-04	2.38E+00
ZNF8-DT	4.20E-04	2.99E+00
ZNF799	4.30E-04	2.15E+00
ENSG00000283189	4.37E-04	2.47E+00
MEIS1	4.37E-04	2.04E+00

ZNF862	4.53E-04	2.85E+00
DECR2	4.56E-04	2.79E+00
CYP4V2	4.56E-04	2.12E+00
ZNF441	4.58E-04	2.29E+00
ZSCAN5A	4.59E-04	2.41E+00
GSTA4	4.60E-04	2.06E+00
LRP5L	4.65E-04	2.28E+00
GLB1L	4.65E-04	2.41E+00
NOTCH2NLB	4.71E-04	2.21E+00
BMPR1B	4.73E-04	2.54E+00
ITPR1	4.74E-04	3.17E+00
INSR	4.86E-04	6.06E+00
FAM27C	4.89E-04	3.03E+00
SH3RF3	4.97E-04	2.06E+00
LCMT2	5.01E-04	2.44E+00
ALDH3A1	5.03E-04	3.55E+00
LRRC27	5.06E-04	2.47E+00
ZNF613	5.10E-04	2.16E+00
CSGALNACT1	5.13E-04	2.47E+00
MTARC1	5.13E-04	2.11E+00
MIR600HG	5.17E-04	2.45E+00
KCTD14	5.19E-04	2.48E+00
SMIM10L1	5.21E-04	2.31E+00
ZBED8	5.32E-04	2.06E+00
FBXO36	5.44E-04	2.80E+00
EXOG	5.47E-04	2.62E+00
MMAA	5.54E-04	2.59E+00
ZNF767P	5.71E-04	2.07E+00
TCAIM	5.72E-04	2.43E+00
DYNLT2B	5.72E-04	2.40E+00
PELI2	5.82E-04	2.12E+00
ABRAXAS1	5.97E-04	2.18E+00
GPC2	6.12E-04	2.82E+00
MORN1	6.34E-04	2.96E+00
MZF1-AS1	6.38E-04	3.21E+00
FIZ1	6.55E-04	2.22E+00
HERPUD2-AS1	6.74E-04	2.23E+00
BORCS6	6.76E-04	2.34E+00
ZNF527	6.80E-04	3.17E+00
KIAA1549	6.86E-04	2.05E+00

MTHFD2L	6.89E-04	3.58E+00
PCDHB14	6.97E-04	2.48E+00
SMARCD3	7.07E-04	2.64E+00
FBLN5	7.13E-04	2.54E+00
ENSG00000272141	7.17E-04	3.19E+00
WDPCP	7.25E-04	2.50E+00
TTC30B	7.36E-04	2.12E+00
RNFT2	7.41E-04	2.10E+00
ARNTL	7.43E-04	2.05E+00
C2orf74	7.49E-04	2.08E+00
ENSG00000217801	7.51E-04	3.28E+00
GIN1	7.57E-04	2.52E+00
HOXC6	7.61E-04	2.02E+00
FANCM	7.73E-04	2.07E+00
ARHGAP11B	7.81E-04	2.96E+00
ZNF425	8.01E-04	2.53E+00
NOXA1	8.09E-04	2.83E+00
DGAT2	8.15E-04	2.61E+00
ENSG00000265690	8.28E-04	2.44E+00
ALKBH8	8.53E-04	2.22E+00
MAST1	8.54E-04	2.81E+00
PPIAP31	8.68E-04	2.75E+00
SORBS1	8.69E-04	2.30E+00
HOXA-AS2	8.76E-04	2.03E+00
ENSG00000256646	8.88E-04	2.50E+00
KCNC4	9.07E-04	2.44E+00
ZNF271P	9.08E-04	2.39E+00
SMPD1	9.39E-04	2.06E+00
EDN1	9.48E-04	3.58E+00
ERICH5	9.59E-04	2.06E+00
MESP1	9.60E-04	3.34E+00
SPRYD4	9.61E-04	2.43E+00
TBCE	9.77E-04	2.82E+00
H2AC18	9.91E-04	3.05E+00
ZNF688	1.01E-03	2.02E+00
ZNF485	1.04E-03	2.37E+00
C15orf62	1.04E-03	2.86E+00
EBF4	1.05E-03	2.83E+00
ENSG00000273066	1.06E-03	2.12E+00
CCT6P3	1.10E-03	2.72E+00

MAP10	1.13E-03	2.23E+00
ANKRD34A	1.14E-03	2.51E+00
DNLZ	1.14E-03	2.04E+00
SLC25A21	1.14E-03	2.56E+00
TBC1D22A-DT	1.16E-03	3.52E+00
LINC01881	1.17E-03	2.25E+00
DLG4	1.20E-03	2.18E+00
SH3BP5-AS1	1.21E-03	2.50E+00
LMNTD2	1.21E-03	2.39E+00
GDPD1	1.24E-03	2.03E+00
NPAS2	1.26E-03	3.70E+00
IDUA	1.28E-03	2.03E+00
ENSG00000285796	1.29E-03	2.36E+00
ENSG00000247679	1.29E-03	2.66E+00
C1QL1	1.29E-03	2.02E+00
SLF1	1.37E-03	2.04E+00
FAM161A	1.39E-03	2.10E+00
CCDC18	1.40E-03	2.12E+00
C5	1.46E-03	2.23E+00
ENSG00000286431	1.46E-03	2.23E+00
YAE1	1.46E-03	2.08E+00
ZBTB26	1.46E-03	2.08E+00
ENSG00000235859	1.48E-03	2.15E+00
PLA2G6	1.48E-03	2.16E+00
CCDC107	1.49E-03	2.13E+00
APBA1	1.49E-03	2.93E+00
MEF2B	1.52E-03	2.90E+00
ZNF678	1.55E-03	2.10E+00
ENSG00000272884	1.70E-03	2.54E+00
EPHA4	1.74E-03	3.10E+00
CLK1	1.74E-03	2.55E+00
TRIM73	1.75E-03	2.32E+00
ADAM22	1.79E-03	3.09E+00
ZNF583	1.80E-03	2.30E+00
LINC01588	1.80E-03	2.28E+00
PBX3	1.83E-03	2.03E+00
ZNF566-AS1	1.88E-03	2.64E+00
RMDN2	1.92E-03	2.08E+00
ENSG00000286129	1.95E-03	2.06E+00
ZSCAN22	2.04E-03	2.18E+00

ENSG00000269044	2.06E-03	2.04E+00
RAB30-DT	2.10E-03	2.02E+00
ZNF619	2.11E-03	2.24E+00
CCDC22	2.15E-03	2.24E+00
RIMS2	2.20E-03	2.81E+00
LINC01270	2.24E-03	2.06E+00
SDHAP3	2.27E-03	2.88E+00
SLC27A5	2.33E-03	2.39E+00
PDPR2P	2.35E-03	2.02E+00
ZNF708	2.46E-03	2.09E+00
MAML3	2.50E-03	2.37E+00
TIGD3	2.51E-03	2.27E+00
ZNF620	2.56E-03	2.13E+00
STAG3L5P	2.58E-03	2.07E+00
CGNL1	2.63E-03	2.64E+00
ENSG00000279047	2.77E-03	8.38E+00
ZNF20	2.81E-03	3.08E+00
HSD17B6	2.86E-03	2.23E+00
IQCH-AS1	2.93E-03	2.05E+00
SBF2-AS1	3.13E-03	2.14E+00
ENSG00000241489	3.15E-03	2.07E+00
SOWAHB	3.26E-03	2.81E+00
FMO4	3.40E-03	2.16E+00
POLR3G	3.43E-03	2.19E+00
KRT23	3.50E-03	3.33E+00
SHANK2	3.60E-03	2.15E+00
SMG1P7	3.65E-03	2.33E+00
ZCWPW1	3.72E-03	2.24E+00
ENSG00000288851	3.74E-03	2.20E+00
ENSG00000283761	3.83E-03	2.44E+00
DUSP19	3.98E-03	2.49E+00
PLAAT3	4.07E-03	2.38E+00
ZNF232-AS1	4.12E-03	2.16E+00
ZNF503-AS2	4.34E-03	2.57E+00
TMEM17	4.48E-03	2.25E+00
ZNF460	4.77E-03	2.10E+00
APBB1	5.14E-03	2.88E+00
AADAC	5.41E-03	2.28E+00
DISC1	5.42E-03	2.33E+00
ENSG00000271793	5.67E-03	3.41E+00

PTP4A3	5.68E-03	2.01E+00
OVGP1	5.77E-03	2.32E+00
MDP1	6.05E-03	2.82E+00
NIFK-AS1	6.06E-03	2.10E+00
PDCD6P1	6.09E-03	2.02E+00
ST20	6.11E-03	2.08E+00
ENSG00000275234	6.17E-03	2.01E+00
ENSG00000289100	6.58E-03	3.04E+00
ENSG00000215068	7.82E-03	2.05E+00
TUBBP1	7.93E-03	2.20E+00
PNMA2	8.31E-03	2.76E+00
ULBP1	8.59E-03	2.02E+00
ZNF345	8.94E-03	2.09E+00
KRT8P3	9.02E-03	2.47E+00
SMIM11	1.04E-02	2.26E+00
ENSG00000280385	1.05E-02	2.12E+00
CMPK2	1.07E-02	3.36E+00
ENSG00000272916	1.28E-02	2.12E+00
FRAT1	1.43E-02	2.31E+00
NEURL3	1.46E-02	2.38E+00
RPL10P16	1.91E-02	2.35E+00
ENSG00000275807	2.05E-02	2.08E+00
MTCO2P12	2.25E-02	2.03E+00
ZNF8-ERVK3-1	2.37E-02	2.02E+00
MTCO3P12	2.93E-02	2.16E+00
MTND4P12	3.16E-02	2.14E+00
ZNF709	4.00E-02	3.65E+00
ENSG00000273590	4.11E-02	2.02E+00
ENSG00000269693	4.45E-02	2.52E+00
