# Supplementary material for: VprBP/DCAF1 Triggers Melanomagenic Gene Silencing through Histone H2A Phosphorylation
Source: Biomedicines. 2023 Sep 17;11(9):2552. doi: 10.3390/biomedicines11092552 (PMC10526264; doi:10.3390/biomedicines11092552)
Supplement: Supplementary file 1 [file biomedicines-11-02552-s001.zip › Information S2-Shin et al.pdf]

List of enriched Gene Ontology (GO) terms for the up-regulated genes from VprBP knockdown cells.

GO:Biologycal Process

ID	GeneRatio	pvalue	p.adjust	qvalue	Count
GO:0044281	139/963	1.17E-06	4.08E-03	4.08E-03	139
GO:0006082	83/963	1.76E-06	4.08E-03	4.08E-03	83
GO:0043436	82/963	2.46E-06	4.08E-03	4.08E-03	82
GO:0019752	77/963	1.71E-05	2.12E-02	2.12E-02	77

GO:Cellular Component

ID	GeneRatio	pvalue	p.adjust	qvalue	Count
GO:0005739	138/1008	3.53E-09	2.17E-06	2.15E-06	138
GO:0019866	51/1008	3.98E-05	1.22E-02	1.21E-02	51
GO:0031966	62/1008	8.57E-05	1.76E-02	1.74E-02	62
GO:0005743	45/1008	1.33E-04	2.05E-02	2.03E-02	45
GO:0005759	43/1008	2.57E-04	2.91E-02	2.89E-02	43
GO:0005740	63/1008	2.84E-04	2.91E-02	2.89E-02	63

GO:Molecular Function

ID	GeneRatio	pvalue	p.adjust	qvalue	Count
GO:0003700	165/1013	1.09E-20	1.01E-17	9.75E-18	165
GO:0000981	159/1013	8.40E-20	3.89E-17	3.76E-17	159
GO:0000977	160/1013	1.04E-18	3.20E-16	3.09E-16	160
GO:0003690	179/1013	1.40E-18	3.24E-16	3.13E-16	179
GO:1990837	171/1013	2.24E-18	4.16E-16	4.02E-16	171
GO:0043565	177/1013	8.60E-18	1.14E-15	1.11E-15	177
GO:0000976	165/1013	8.71E-18	1.14E-15	1.11E-15	165
GO:0001067	165/1013	9.88E-18	1.14E-15	1.11E-15	165
GO:0140110	186/1013	8.42E-15	8.67E-13	8.38E-13	186
GO:0000978	120/1013	1.52E-10	1.40E-08	1.36E-08	120
GO:0000987	120/1013	4.41E-10	3.71E-08	3.59E-08	120
GO:0016788	72/1013	3.40E-06	2.63E-04	2.54E-04	72
GO:0030170	12/1013	3.47E-05	2.48E-03	2.39E-03	12
GO:0070279	12/1013	4.20E-05	2.78E-03	2.69E-03	12
GO:0001227	35/1013	1.01E-04	6.23E-03	6.02E-03	35
GO:0001217	35/1013	1.29E-04	7.46E-03	7.21E-03	35
